# Meeting Reports

**DOI:** 10.1155/S1463924680000338

**Published:** 1980


### Meeting Reports

### 31st Pittsburgh Conference

The 31st Pittsburgh Conference of Analytical Chemistry and Applied Spectroscopy was held in Atlantic City, N. Jersey, USA, 10–14 March 1980.

Despite the many problems associated with Cleveland as the venue for the Pittsburgh Conference it had become accepted and it seemed strange this year breaking off one's journey to Cleveland at New York and travelling down to a new venue at Atlantic City. However, the 31st "Pittsburgh Conference" continued in fine tradition and from all aspects: numbers of papers, exhibitors and attendees, increased in size compared with previous conferences. It is an extraordinary occasion providing a unique opportunity to meet almost everyone of note and to view new equipment for the first time. The various symposia and conference sessions also provide an opportunity for many people to lecture for the first time, and for instrument companies to present their new products for the market. Sadly, not all of the lectures were of a really high standard.

This year there were more than 800 individual presentations and significantly about 10% of these had some automation content. The proceedings of this conference are not published 'en bloc' but most authors will provide further details or scripts for those interested.

The title of the first automation session was "Flow Injection and Automatic Analysis I". The session was well attended, but when any of the more difficult aspects such as sample preparation were discussed, it was clear that the audience thinned out. Many people have yet to grasp the fundamentals of automatic analysis and they view FIA as a cheap alternative to the more costly automated analysis systems. Of course, FIA has a role to play in the field of automation and it does provide an entree into practical experience, but attention to the basic philosophy, management and economics of the subject must be continued.

Schick [1] described a novel system for determining free and combined chlorine using the kinetics of the oxygen peroxide decomposition reaction and subsequently estimating the analyses using an amperometric oxygen probe. A chlorine flow injection analyzer was described which was capable of maintaining anaerobic conditions. After injection of 50 $\mu$l of hydrogen peroxide into the sample stream, the localised $pO_2$ increase is measured by the oxygen probe in a flow through cell. The addition of potassium iodide to the injected stream modifies the reaction conditions such that monochloramine and dichloramine can be differentiated. Detection limits of 0.30 ppm with an upper limit of 75 ppm were reported, the latter being limited by oxygen saturation in the reagent stream.

Karlbert [2] described a flow injection analysis system for the analysis of ammonium ions at the sub ppm levels. The system utilises a dialysis cell fitted with a teflon membrane. The sample is injected as a 30 litre plug. It is mixed with NaOH and the ammonium ions generate NHz which diffuses through the membrane into a recipient stream containing a small amount of phenol red acid base indicator. Results from a wide variety of samples such as Kjeldah digests, blood serum, whole blood, waste water and amino acid solutions were presented. A detection limit of 0.02 ppm at the rate of 60 samples per hour was obtained.

Stewart [3] one of the co-inventors of this flow injection technique described further extensions of his work on the automated multiple flow injection analysis system (AMFIA). He has extended it to provide more automation and also to allow operators either at a research or at a routine supervisory level to interact with the system to update the operating parameters, to include new analytical operations or to modify calculation methods. Programs and data for the system can either be stored in cassette tapes or an additional central computer. A Rockwell AIM 65 micro-computer with 20-column thermal printer, 20 character alphanumeric display, full ASCII keyboard and an 8K ROM BASIC provide the main elements of the control and data system. A 12 bit A/D convertor and low impedance operational amplifier were used for data acquisition. One of the main aims of the software is to allow the user to be fully interactive with the automation without detailed knowledge of assembly or BASIC programming. Six program words have been defined to allow the user to perform complete functions related to AMFIA situations. A sound background to the system design was presented which takes the FIA some way to becoming an automated technique of importance rather than an academic curiosity.

Ranger [4] contrasted flow injection analysis with liquid chromatography and described the interfacing of the latter technique with a polarographic detector. The primary advantage in so doing was the provision of a fast automatic and reproducible sample pre-treatment and feeding mechanism for the detection system. Classical bubble segmented streams are not as adaptable to polarographic detection. One major area of application, the plating industry, was described and to date copper, nickel, lead and tin have been successfully analysed using the approach described. He predicted that future areas of interest would include pharmaceutical and water analysis.

Brown [5] whose work on sample preparation has been described in this journal previously (*The Journal of Automatic Chemistry* 1980, 2 15.) provided further evidence of the ingenuity of staff at his laboratory, when he described in detail a semi-automated ultrasonic food extraction system. Under microprocessor control, samples are ultrasonically disintegrated and extracted by one or more solvents. Sample residues are then purged from the system and collected for further analysis. Cleaning of the system, often a time-consuming and difficult process in manual analysis, is also readily carried out by ultrasonic action. This semi-automatic technique accurately represents the manual protocols laid down for analysis. However it reduces the operator's involvement by some 90%, thereby effecting a considerable saving in manpower.

High resolution gas chromatographic analysis now allows a true peak by peak analysis to be effective in contrast to presently accepted empirical methods. However, the multiplicity of peaks and the partial fusion of some of them presents a difficult task to the state-of-the-art integrators, which are indispensible when such a large volume of data is generated. Problems relating to this aspect of data analysis were discussed by Snow [6] in relation to chromatograms of PCB's. Three integrators were compared in the study; the Hewlett Packard 5840 and the 3385A systems along with the Spectra Physics SP 4100 computing integrator. Chromatograms of four commercially available Anochlar mixtures were presented in the paper and the results from each of the integrators compared.

Klein [7] described a system used for the analysis of inhalation chambers concentrations of elthyric oxide. The system used improvised a Perkin Elmer automatic sampling system, a gas chromatograph with flame ionisation detector, an integrator, and analogue recorder and a cathode ray data terminal equipped with a dual magnetic tape recorder and printer. Data recorded on the system were then transferred to a PDP-II computer for tabulation, statistical analysis and archiving. Results of a two year study were then presented.

In the final paper of this session Vanderslice [8] set out a theoretical framework to describe FIA. Previous work defining the technique has been expanded to cover a wide range of variables which include all those of experimental interest for flow injection analysis. Predictions of curve shape, maximum sensitivity, height and area measurement and analysis times are discussed as a function of flow rate, tubing diameter, sample size, reaction volumes and diffusion coefficient.

In the first paper of the second automation session entitled "Automated Analysis II" Balciunas [9] described a microcomputer-controlled, modular, syringe-based reagent preparation system. Stock reagents are delivered from each of three stepper motor driven syringes through a 6 way selector valve into a volumetric dilution vessel. The diluent is then drawn through the selector valve into the volumetric vessel until the solution reaches an optical limit detector. Appropriate valves are then switched to deliver the diluted solution to any container. The ability of the system to accurately deliver small volumes was demonstrated using a continuous variation study of a 3.1 completion reaction. The reagents can be reliably diluted over a range of 5 orders of magnitude. The use and reproducibility of the system were also discussed in some detail.

Snider [10] discussed an evaluation of the Du Pont preparture system for preparation of serum samples in bioavailability studies in a pharmaceutical laboratory. A wide range of dosage forms were also studied. The preparative system increased the precision of the serum assay by decreasing the between-run variation. The manpower for the analysis was also significantly reduced. Several applications were described to illustrate the general applicability of the unit for solvent extractions.

Dugger [11] presented a computer program to correct mass spectra recorded from a quadruple spectrometer. The program can assess existing data files, read the data into an array, modify the array using the correction factors and write the corrected data onto a new file. Typical illustrations were presented.

Knopf [12] presented results of a comparative study of the Leco NP-28 nitrogen protein analyser against the conventional protein method. A sample in a given matrix was analysed in duplicate on eight consecutive days by both methods. Matrices analysed included soya bean meal feed, feed ingredients, pet foods and faeces.

Weinberger [13] described the application of an infrared detector in association with an AutoAnalyzer system used to measure simethicone (polydimethyl – silox) in pharmaceutical solid dosage forms. The simethicone is extracted with 70 ml kerosene and 35 ml of 5% concentrated HCl. The upper kerosene layer is aspirated filtered and passed through a calcium fluoride flow cell. A sharp absorption maximum for silicone polymers occurs at 7.9 $\mu$. Twenty samples per hour are analysed with a precision of better than 4%. Intersample carry-over of the order of 3% is obtained and the recovery approaches 100%.

Iorns [14] then described a fully automatic computer-controlled instrument capable of the simultaneous determination of calcium, hydrogen, nitrogen and sulphur in a wide variety of materials. The instrument operates in the following manner: a sealed sample is dropped into a quartz combustion tube held at 1025 °C in the presence of oxygen, the combustion gases are passed through a reduction tube containing a copper reducer at 850 °C and then separated and analysed using a unique three column gas chromatographic system. Techniques for handling a wide variety of samples were described and typically standard deviations of around 0.11 for carbon, 0.06 for hydrogen, 0.09 for nitrogen and 0.16 for sulphur were obtained.

Wolf [15] discussed the further extension of AMFIA to provide automated simultaneous multielement flame atomic absorption using a continuous source. A combined system was described which analyses up to 16 elements at a rate of 60 samples per hour and allows the use of extended dynamic range techniques to obtain linear dynamic ranges of 4 – 5 orders of magnitude. A study of the detection limits and the necessary compromises for routine operation were also discussed.

The determination of potassium in a liquid process stream by measuring the naturally radioactive $^{40}$K was described by Langhorst [16]. The 1.46 mev gamma ray associated with $^{40}$K is detected by a NaI (TL) scintillation detector. This avoids interference from members of the uranium and thorium series. An Intel 80/20 microcomputer is used to collect and correlate the data and to display the results. Better process control is provided to the plant operating personnel using the system described.

In the final paper Duchane [17] described the use of a highly automated Inductively Coupled Plasma system for the multielemental analysis of NURE water samples. Twelve elements are measured simultaneously using a Plasma-Therum ICP in conjunction with a Jarrell Ash spectrograph with a direct reader attachment. Data analysis is performed using a DEC PDP 11/20 computer. Automated sample preparation is effected using a Gilson autosampler, this also acts as the master controller to direct all phases of the operation of the analytical system and allows completely unattended operation. The operation of the system was described in some detail.

Many more of the papers, as mentioned earlier, related to automatic analysis. Also more aspects of the instrumentation shown in the exhibition truly reflect automation. In most cases this relates to the control and data handling aspects of instruments; as yet there is still a lack of completely automated systems which include sample preparation. Surprisingly little new instrumentation was on show – the real quantum jump using microprocessors occurred a few years ago. However, now the power of the micros is being further utilised and the instruments encompass many more sophisticated and useful facilities. The use of computer graphics is increasing slowly despite its obvious advantages in displaying large volumes of data in a manageable form. Some interesting products introduced or shown at the conference are described on pages 101–109 of this issue.

*Peter B. Stockwell*

BIBLIOGRAPHY

[1] Flow injection analysis of free and combined chlorine utilizing a novel oxygen evolution reaction – K.G. Schick, Fiatron Systems Inc, 7315 First Call Creek, WI 53154 USA.
[2] Flow-injection analysis of ammonium ions at sub ppm levels – B.I. Karlberg. T. Bifok AB, Box 5024, S-194 05, Uplands-Vasby, Sweden.
[3] Microcomputer control of automated multiple flow injection analysis (AMFIA) – K.K. Stewart, United States Department of Agriculture, Beltsville, MD20705, USA.
[4] Automatic flow injection polarography for laboratory and on-line determinations of metals and organics – C.B. Ranger, Lachat Instruments Inc, 10500 N. Port, Washington Road, Mequor, WI 53092, USA.
[5] A semi-automated ultrasonic food extraction system – J.F. Brown, United States Department of Agriculture, Beltsville, MD20705, USA.
[6] Use of a microprocessor integrator for analysis of samples containing complex mixtures of PCB by glass capillary chromatography – J. Snôw, New York State Health Dept., Empire State Plaza, Albany, New York 12201, USA.
[7] An automatic gas chromatographic system for ethylene oxide inhalation chamber analysis – G.W. Klein, Carnegie Mellon University, Mellon Institute, 4400 5th Avenue, Pittsburgh, PA152, USA.
[8] Laminar dispersion theory applied to flow injection analyses – J.T. Vanderslice, United States Department of Agriculture, Beltsville, MD20705, USA.
[9] An automated reagent preparation system – R.T. Balciunas, Michigan State University Dept. of Chemistry, Lansing. Mi 48824, USA.
[10] Automation of sample extractions with the DuPont Prep – B.G. Snider, the Upjohn Company, Kalamazoo, MI 49001, USA.

[11] A computer program for correcting quadruple mass spectra from GC/MS data – D.L. Dugger, GTE Labs, Materials Evaluation, 40 Sylvian Road, Waltham, MA 02154, USA.

[12] LECO NP-28 nitrogen protein determinator vs. Kjeldahl protein method – M.F. Knopf, Raltech Scientific Services, 900 Checkerboard Square, St. Louis, MO 63188, USA.

[13] Automated analysis of simethicone in tablets by infrared spectroscopy – R. Weinberger, Reed & Carnrick Research Inst., Keniworth, N. Jersey 07033, USA.

[14] An automated analyzer for the simultaneous determination of carbon, hydrogen, nitrogen and sulphur in coal and other organic and inorganic materials – T.V. Iorns, Phillips Petroleum Company, Research & Development Dept., RB/1 Bartlesville, OK 74004, USA.

[15] Automated simultaneous multielement flame atomic absorption utilizing flow injection analysis: AMFIA-SIMAAC – W.R. Wolf, United States Department of Agriculture, Beltsville, MD20705, USA.

[16] On-line determination of potassium in a liquid process stream by gamma ray spectroscopy using a Na (TL) detector – M.A. Longhorst, Dow Chemical Company, Analytical Labs, 574 Building, Midland, MI48640, USA.

[17] Highly-automated, operated-unattended, ICP analysis of NURE Water Samples – D.V. Duchane, University of California, Los Alamos Laboratory, Los Alamos, NM 87545, USA.

### Sophistication in instrumentation – has it gone too far?

This discussion meeting organised by the Analytical Division of the Chemical Society, was held at Imperial College, London, on 13 February.

The view that instruments had become too sophisticated was supported by Dr D A Pantony from the Royal School of Mines, London. He agreed that advanced techniques and equipment are needed to deal with difficult analytical problems, but regretted that a great deal of flexibility had been lost in the elaborate equipment on the market. With complex machines, control is taken out of the operator's hands, who may then fail to notice when things go wrong. He also expressed the opinion that there was a tendency for people to want to have the latest instrumentation developments regardless of whether they really need them. Simpler and cheaper equipment may do what is required equally well. In addition, research laboratories, which are in a sense 'non-productive' must be seen to keep costs down to justify their position.

Dr Pantony made a number of recommendations to prospective purchasers. These included comparing the cost of the dedicated computers incorporated into instruments with the use of central computing facilities. He felt that the benefits gained from the inclusion of a microprocessor in some machines was out of proportion with the extra price. People should only buy the basic equipment and not be tempted by the extra facilities introduced on every new model, as these may only rarely be used.

Although Dr Pantony freely admitted that sophisticated instrumentation has advantages for routine analysis, researchers need flexible, basic instruments. There is a decreasing amount of basic equipment on the market, so from his point of view as a researcher, instrumentation has become too sophisticated.

Dr J E Cantle from Instrumentation Laboratories Ltd, Altringham, UK, answered Dr Pantony's arguments against by giving the manufacturers' point of view. He said that there is no doubt that complex instruments are needed to cope with modern analysis, and that sophistication had improved the scope of instruments. By producing such equipment, manufacturers are simply responding to the demand from customers. Dr Cantle admitted that in many cases new facilities are added to instruments to beat competitors, but justified this by saying that such developments are only incorporated when market research shows that there was a need for them. He denied that there is always an associated price increase with the addition of new facilities, but had to admit that this was usually the case. If a new instrument which was too sophisticated for the market, was launched however, it would not be successful. Dr Cantle agreed that the increasing complexity of instruments does make it difficult for prospective buyers with a specific problem to solve, to decide which of the available techniques, accessories and facilities are essential to his requirements.

In response to Dr Pantony's comments, Dr Cantle argued that the system would only be at fault if the basic instruments were no longer available.

In the open discussion that followed a number of interesting questions were raised. Problems often arise because the people actually buying the instruments did not know the pros and cons of the various facilities offered, and because it is not always possible to guarantee in advance what types of analysis will be required in the future, so that the right instrument can be chosen.

A number of people expressed the belief that microprocessors were often thrown in by the manufacturers as a gimmick to make the instrument appear more 'up market' and therefore more expensive. It was felt that if the instrument incorporated a microcomputer, that this should be used as more than just a calculator, as in many cases, but should provide fault finding and variable parameter facilities.

The question 'but what happens if it goes wrong?' is one that worries many buyers. Dr Cantle commented that prospective purchasers frequently asked if they could use the machine manually if it broke down. In many case the instruments are so complex that only a specially trained person can repair them. The time is coming when every laboratory will need an electronics engineer just to look after the instruments. Concern was shown that not only will the operator be unable to repair the machine, he will probably not have the experience to recognise that something is wrong in the first place. This creates a general lack of confidence in the instruments results, tying up the very staff whose time the machine was supposed to save in testing the instrument's accuracy and supervising its operation.

The difficulties of instrument evaluation before purchase and the problems encountered by the manufacturer in finding a compromise level of sophistication to satisfy the largest proportion of users were also discussed.

At the close of the meeting it appeared to be agreed that the equipment currently available was not sophisticated enough for the routine analysis market (where the manufacturer can expect to make his profit) because most of the instruments do not have efficient fault finding capabilities. However, the available instruments are too sophisticated for research which requires basic equipment.

*Lesley Colgate*

